# 

**Published:** 1890-01

**Authors:** 


					﻿JAISFU’.AJRY, 1890.
OLD SERIES
Vol. XXV
NEW SFFVfcS
Vol. VI.-No. II, j,
1855 <>

Wedigal^wK
(Journal


Sk
W. F. WESTMORELAND, M.D.
H. V. M. MILLER, M.D., LL.D.
VIRGIL 0. HARDON, M.D.
F. W. McRAE, M.D., Man. Editor.
Published by james p.harrison&cqe
ff___________ — t&TLANTA,GA. Jit
SUBSCRIPTION t 12.00 A YEAR
				

